# The promotion effect of manganese on Cu/SAPO for selective catalytic reduction of NO_*x*_ with NH_3_

**DOI:** 10.1039/c7ra12350g

**Published:** 2018-02-06

**Authors:** Chengkai Pang, Yuqun Zhuo, Qiyu Weng, Zhenwu Zhu

**Affiliations:** Key Laboratory for Thermal Science and Power Engineering of Ministry of Education, Beijing Engineering Research Center for Ecological Restoration and Carbon Fixation of Saline-alkaline and Desert Land, Department of Thermal Engineering, Tsinghua University Beijing 100084 China zhuoyq@tsinghua.edu.cn

## Abstract

The activity and hydrothermal stability of Cu/SAPO and *x*Mn–2Cu/SAPO for low-temperature selective catalytic reduction of NO_*x*_ with ammonia were investigated. An ion-exchanged method was employed to synthesize *x*Mn–2Cu/SAPO, which was characterized by N_2_ adsorption, ICP-AES, X-ray diffraction (XRD), NH_3_-temperature programmed desorption (NH_3_-TPD), NO oxidation, X-ray photoelectron spectrum (XPS), UV-vis, H_2_-temperature programmed reduction (H_2_-TPR) and diffuse reflectance infrared Fourier transform spectra (DRIFTS). 2Mn–2Cu/SAPO and 4Mn–2Cu/SAPO showed the best SCR activity, in that at 150 °C NO conversion reached 76% and N_2_ selectivity was above 95% for the samples. NO oxidation results showed that the 2Mn–2Cu/SAPO had the best NO oxidation activity and the BET surface area decreased as manganese loading increased. XRD results showed that the metal species was well dispersed. NH_3_-TPD showed that the acid sites have no significant influence on the SCR activity of *x*Mn–2Cu/SAPO. H_2_-TPR patterns showed good redox capacity for *x*Mn–2Cu/SAPO. UV-vis and H_2_-TPR showed that the ratio of Mn^4+^ to Mn^3+^ increased as manganese loading increased. XPS spectra showed a significant amount of Mn^3+^ and Mn^4+^ species on the surface and addition of manganese increased the ratio of Cu^2+^. The promotion effect of manganese to 2Cu/SAPO comes from the generation of Mn^3+^ and Mn^4+^ species. Deduced from the DRIFTS spectra, the Elay–Rideal mechanism was effective on 4Mn–2Cu/SAPO.

## Abstract 2

The activity and N_2_ selectivity of Cu/SAPO and *x*Mn–2Cu/SAPO for low-temperature selective catalytic reduction of NO*_x_* with NH_3_ were investigated.

## Graphical abstract

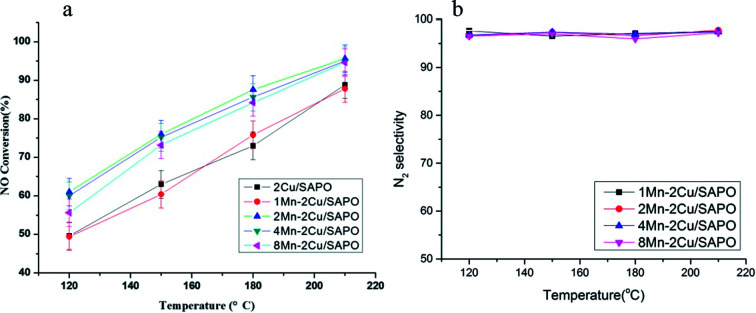

## 1 Introduction

NO_*x*_ consists of NO, NO_2_, N_2_O and their related derivatives, which can lead to numerous environmental and health hazards.^1^ By forming nitric acid, NO_*x*_ can lead to the generation of acid rain; by forming ammonium nitrate, it can accelerate the generation of PM2.5; by reacting with hydrocarbons under sunlight, it can cause the generation of photo chemical smog and ozone; by infiltrating into lungs, it can result in respiratory morbidity, like impaired host defense and lung inflammation. Thus, legislations on NO_*x*_ emission are now becoming more and more stringent. In China, according to the emission standard of air pollutants for thermal power plants (GB 13223-2011), the NO_*x*_ emission limit is 100 mg m^−3^ for new natural gas-fired industrial boilers and all oil and coal-fired industrial boilers. For compression ignition and gas fueled positive ignition engines of vehicles, the NO_*x*_ emission limit in Chinese National 5 standards is 57% of that in Chinese National 4 standards and 40% of that in Chinese National 3 standards.

The treatment of environmentally harmful NO_*x*_ compounds emitted from mobile or stationary sources remains a challenging task, especially for industrial applications such as cement plants or iron and steel plants, because the suitable process temperature for NO_*x*_ abatement is usually around 150 °C, under which commercial SCR catalysts V_2_O_5_–MoO_3_ (WO_3_)/TiO_2_ cannot work well.

Manganese oxides were reported by Smirniotis *et al.* as potential catalysts for low-temperature NH_3_-SCR reactions.^2–5^ They studied the promoted manganese oxides supported on TiO_2_ systematically^3–5^ and found that the surface Mn^4+^ species was reported to be highly active for the SCR of NO reaction with ammonia at low temperatures.

Recently, copper modified zeolites with a CHA structure attracted much attention owing to their excellent activity, N_2_ selectivity and hydrothermal stability for low temperature NH_3_-SCR reaction.^6–8^

However for the Cu/SAPO-34 catalyst, the oxygen activation of transiently formed Cu pairs [Cu^I^(NH_3_)_2_] to [(NH_3_)_2_Cu^II^-O_2_-Cu^II^(NH_3_)_2_] was rate-limiting in the catalytic cycle.^8^ Feng Gao^9^ also thought that the oxidation reaction of Cu^+^ to Cu^2+^ was the rate-determine step, so tuning the redox properties of the active site by introducing a second cation might be a promising approach. Li *et al.*^10^ and Chen *et al.*^11^ modified the Cu/SAPO catalyst with CeO_*x*_ for SCR reaction; they found that the introduction of CeO_*x*_ increased the SCR activity at high temperatures and cerium helped increase the amount of isolated Cu^2+^ ions.

Leistner *et al.*^12^ found that Cu in Cu/SAPO-34 was more easily reduced compared to Cu/SSZ-13, which could facilitate the redox processes and increase the SCR activity. Ma *et al.*^13^ also found that Cu-SAPO-34 showed higher DeNO_*x*_ catalytic activity than Cu-SSZ-13. The usage of SAPO-34 in low-temperature SCR might be favored in low temperature SCR reactions. When manganese is introduced into the Cu/SAPO system, it not only promotes the oxidation reaction of Cu^+^, but also acts as an active site for low temperature SCR reaction. Thus the manganese modified Cu/SAPO-34 catalyst would probably show better SCR activity.

In this work, the treatment of exhaust from a gas-fired boiler which contains a high concentration of H_2_O and a very low concentration of SO_2_ was focused upon. *x*Mn–Cu/SAPO catalysts with different manganese loadings were prepared and their performance in SCR reaction was discussed. Various characterization methods (XRD, XPS, H_2_-TPR, UV-vis) were used to probe the promotion effect of manganese on Cu/SAPO for low temperature SCR reaction.

## 2 Experimental

### 2.1 Catalyst preparation

*x*Mn–2Cu/SAPO catalysts were prepared by an ion-exchange method. The alkali metals in commercial Na/SAPO-34 powder (Jiangsu XFNANO) would decrease SCR activities, so it was transformed to H^+^/SAPO as follows. Na/SAPO-34 powder was ion exchanged using 11% wt NH_4_NO_3_ (Aladdin, >98.5%) solution whose pH value was adjusted to 3.0–4.0 by 2 M ammonium hydroxide (Aladdin, 25–28%) solution at 80 °C for 4 h. It was then filtered and washed with distilled water three times. Finally, it was dried at 110 °C for 16 h and calcined at 550 °C for 3 h.

*x*Mn–2Cu/SAPO (*x* = 0, 1, 2, 4, 8) was prepared in two steps. Firstly, H^+^/SAPO was mixed with Cu(NO_3_)_2_ (across, >95%) solution at 80 °C for 6 h under vigorous stirring. Then it was dried and calcined at 550 °C for 3 h. In the second step, it was mixed with Mn(NO_3_)_2_ (across, >95%) solution at 80 °C for 6 h under vigorous stirring, and at last it was dried and calcined at 550 °C for 3 h.

### 2.2 SCR activity measurements

Catalytic activity evaluation was carried out using a flow-through powder reactor system equipped with a Fourier transform infrared (FT-IR) spectrometer (THERMO SCIENTIFIC IGS). In order to prevent condensation along upstream tubing, all the gas lines were heated and maintained at 120 °C. The gas mixture was 500 ppm NO + 500 ppm NH_3_ + 3% vol O_2_ balanced with N_2_. The gas hourly space velocity (GHSV) was 65 000 h^−1^ for the standard SCR reaction. Prior to each activity measurement, the catalysts were pretreated at 500 °C for 30 min with 21% vol O_2_/N_2_ flow. Catalytic activities were measured in the temperature range of 120 to 210 °C. The typical time to achieve the steady state was 2.5 h. NO conversion, promotion effect, and (*E*_p_) and N_2_ selectivity were calculated at steady state using the equation below:1
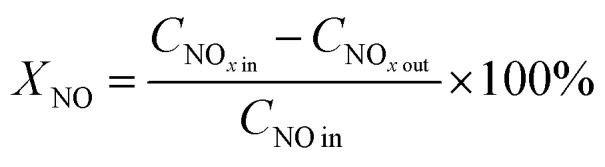

2 *E*_p_ = (*X*_*x*Mn–2Cu/SAPO_ − *X*_2Cu/SAPO_) × 100%

3
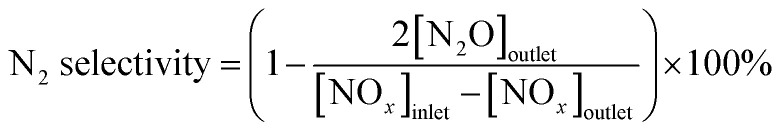
where *C*_NO*x* in_ and *C*_NO*x* out_ represent the inlet and outlet NO_*x*_ concentration. *X*_*x*Mn–2Cu/SAPO_, *X*_2Cu/SAPO_ and *X*_*x*Mn/SAPO_ respectively represent the NO_*x*_ conversion of *x*Mn–2Cu/SAPO, 2Cu/SAPO and *x*Mn/SAPO at 210 °C, and [N_2_O]_outlet_ represents the outlet N_2_O concentration.

### 2.3 Characterization

X-ray diffraction patterns were collected on a bruker D8 Advance X-ray diffractometer with a Ni-Filtered Cu Kα with a step size of 0.02 in the 2*θ* range of 5° to 40°.

Ammonia temperature programmed desorption (NH_3_-TPD) experiments were carried out as follows. 150 mg sample was first pretreated in 21% vol O_2_/N_2_ at 500 °C for 30 min and then cooled to 100 °C in N_2_ and a total flow of 100 ml min^−1^ containing 2500 ppm NH_3_ in N_2_ was injected into the reactor for 2 hours to achieve a steady state. Once the catalyst was saturated, NH_3_ was switched off and the catalyst was swept by N_2_ overnight. Finally, the catalyst was heated in N_2_ at a temperature ramp to 700 °C with a heating rate of 10 °C min^−1^.

NO oxidation experiments were carried out as follows. 150 mg sample was first pretreated in 21% vol O_2_/N_2_ at 500 °C for 30 min and then cooled to 210 °C in N_2_. Then a total flow of 100 ml min^−1^ containing 500 ppm NO and 3% vol O_2_ was injected into the reactor for 30 min to achieve the steady state.

The contents of the elements were determined by ion coupled plasma (ICP) optical emission spectroscopy (Thermo IRIS Intrepid II) after microwave digestion.

The BET surface area, pore volume and pore size of the catalyst samples were measured by N_2_ adsorption using the MICROMERITICS ASAP 2020 surface area and porosity analyzer.

Diffuse reflectance UV-vis spectra were recorded in the range of 200–800 nm against a BaSO_4_ as a reference standard on a HitachiU-3900 UV-vis spectrophotometer equipped with an integration sphere.

H_2_-TPR experiments were performed using 20 mg sample as follows. The catalysts were first pretreated at 500 °C for 30 min in a highly pure O_2_ (40 ml min^−1^) stream. Then the furnace temperature was decreased to room temperature, and feed containing 5% vol H_2_ in N_2_ was fed at a flow rate of 40 ml min^−1^. H_2_-TPR runs were performed as the temperature increased from room temperature to 800 °C at a linear heating rate of 10 °C min^−1^ and then the temperature was kept constant for 30 min at 800 °C to ensure complete reduction. Hydrogen was measured by TCD.

X-ray photoelectron spectroscopy (XPS) analyses were performed using PHI quantera SXM Scanning ESCA Microprobe (Physical Electronics) with a hemispherical detector operating at a constant pass energy (PE = 55 eV). An X-ray source at 210 W (*I* = 15 mA, *U* = 14 kV) and Al Kα radiation (1486.6 eV) were used. Intensities were estimated from the integration of each peak, after smoothening, subtracting the L-shaped background, and fitting the experimental curve to a combination of Lorentzian and Gaussian lines of variable proportions. All binding energies were referenced to the C 1s line at 284.8 eV.

Diffuse reflectance infrared Fourier transform spectra (DRIFTS) were measured on an FT-IR spectrometer (Thermo Nicolet NEXUS870) with an MCT detector and high temperature reaction chamber (Harrick Scientific Praying Mantis) with ZnSe Windows, which was connected to a gas-dosing system. The powder sample was placed in a sample cup and heated by a cartridge heater underneath the sample. The temperature was adjusted using a K-type thermocouple connected to a Harrick temperature controller. Before each measurement, oxidation pretreatments were executed at 500 °C for 1 h. Background spectra were collected before adsorption for 32 scans with a resolution of 4 cm^−1^ in N_2_. DRIFTS spectra were recorded in the range of 4000–650 cm^−1^ for 32 scans with a resolution of 4 cm^−1^.

## 3 Results and discussion

### 3.1 Low temperature NH_3_-SCR activity and N_2_ selectivity

**Fig. 1:**
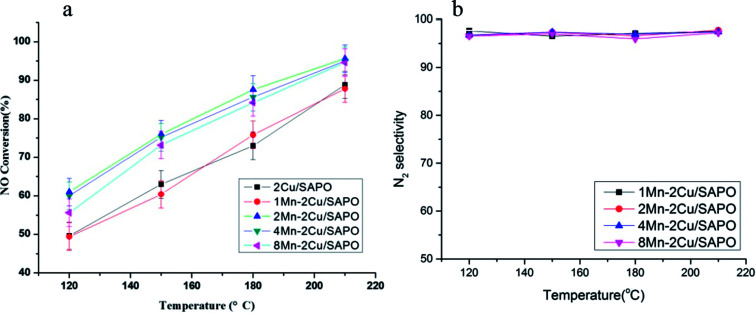
(a) Catalytic activity and (b) N_2_ selectivity of *x*Mn–2Cu/SAPO. Conditions: 500 ppm NO + 500 ppm NH_3_ + 3% vol O_2_ + N_2_ balance, GHSV = 65 000 h^−1^.

Fig. 1a shows the NO conversion of *x*Mn–2Cu/SAPO. There was not much difference in the catalytic activity between 2Cu/SAPO and 1Mn–2Cu/SAPO, 2Mn–2Cu/SAPO and 4Mn–2Cu/SAPO. The activity of 8Mn–2Cu/SAPO was slightly lower than the activity of 2Mn–2Cu/SAPO. As manganese loading increased from 1% to 2%, the activity increased obviously and the SCR activity of 2Mn–2Cu/SAPO, 4Mn–2Cu/SAPO and 8Mn–2Cu/SAPO was much higher than that of 2Cu/SAPO and 1Mn–2Cu/SAPO.

For 2Mn–2Cu/SAPO and 4Mn–2Cu/SAPO, the conversion of NO at 120 °C was about 60%; as the temperature increased to 210 °C, the conversion reached 95%.

Fig. 1b shows the N_2_ selectivity of *x*Mn–2Cu/SAPO. Good N_2_ selectivity was observed, being above 95% for all catalysts. Other researchers^10,11^ also found good N_2_ selectivity for the supported SAPO-34 catalysts.

Compared with *x*Mn/SAPO (*x* = 1, 2, 4, 8), the previously prepared,^14^*x*Mn–2Cu/SAPO showed better SCR activities, especially for 2MN–2Cu/SAPO, indicating that the interactions between copper and manganese might increase its SCR activity.

### 3.2 Water resistance test

**Fig. 2:**
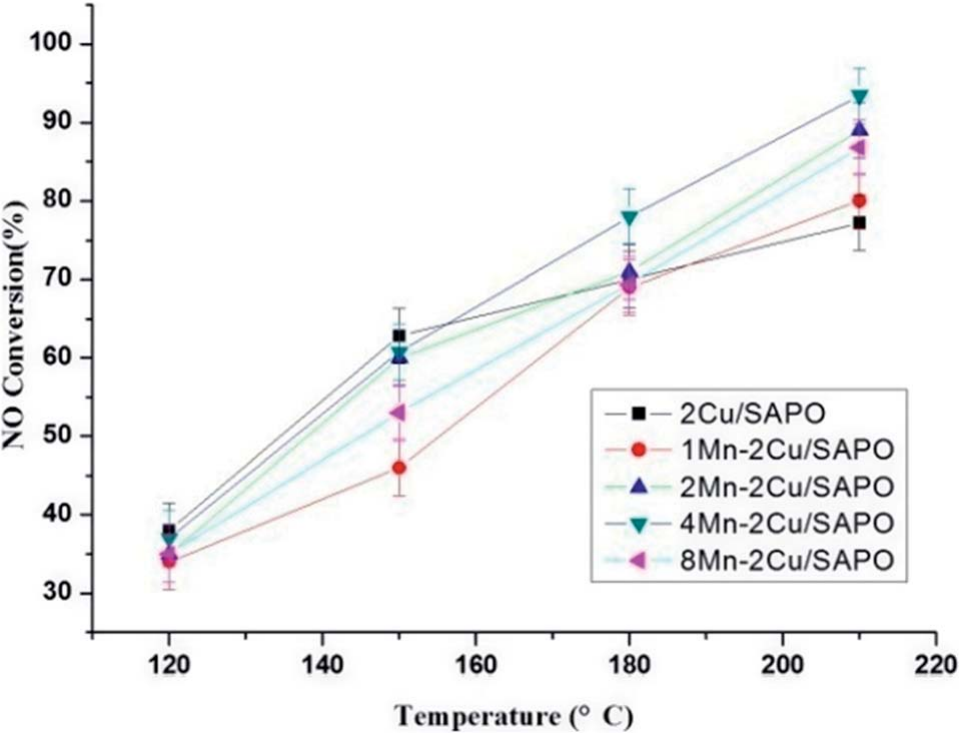
Water resistance test of *x*Mn–2Cu/SAPO for low-temperature SCR. Conditions: 500 ppm NO + 500 ppm NH_3_ + 16.3% H_2_O + 3% vol O_2_ + N_2_ balance, GHSV = 65 000 h^−1^.

Fig. 2 shows the water resistance test of *x*Mn–2Cu/SAPO. The poison effect of water might come from the reversible adsorption of H_2_O^15^ or irreversible dealumination process of zeolite.^16^

In Fig. 2, all catalysts showed strong resistance to water at high temperature. It could be caused by the lower adsorption capability of water at high temperature, since SAPO-34 was highly hydrothermally stable^16^ and the poison effect might originate from the competitive adsorption of water with ammonia.

### 3.3 NO oxidation activity and promotion effect

**Fig. 3:**
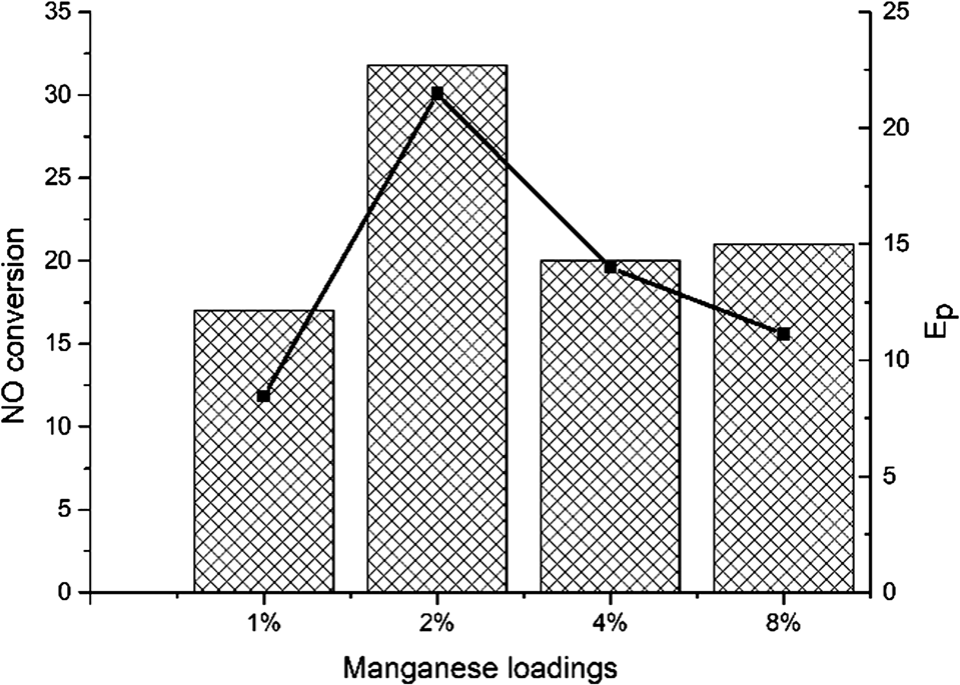
NO oxidation activity and promotion effect of manganese.

Fig. 3 showed the NO oxidation activity of *x*Mn–2Cu/SAPO and the promotion effect. 2Mn–2Cu/SAPO had the best NO oxidation activity, indicating the interaction between copper and manganese elements, which accelerated the NO oxidation process. Good oxidation activity would certainly help the oxidation of Cu^+^ to Cu^2+^ in SCR reaction. The *E*_p_ profile was almost the same as the NO conversion profile, indicating that the increase in NO oxidation activity would promote the low-temperature SCR activity. Compared with the *x*Mn/SAPO (*x* = 1, 2, 4, 8) previously prepared,^14^ the NO oxidation activities of *x*Mn–2Cu/SAPO were much higher, which could be one reason for the increased SCR activity.

### 3.4 Physicochemical properties

As showed in Table 1, the BET surface area and micro pore volume decreased gradually as manganese loading increased; the surface areas of 2Mn–2Cu/SAPO, 4Mn–2Cu/SAPO and 8Mn–2Cu/SAPO were similar. *x*Mn/SAPO (*x* = 1, 2, 4, 8) previously prepared^14^ showed the same trend: the surface area and pore volume decreased as manganese loading increased.

**Table 1:** Physico-chemical properties

Sample	BET surface area (m^2^ g^−1^)	Micro pore volume (cm^3^ g^−1^)	Micro pore width (nm)	Mn loading^a^ (wt%)	Cu loading^a^ (wt%)	Mn/Cu molar ratio^a^	Mn/Cu molar ratio^b^
2Cu/SAPO	467	0.231	2.36		1.951		
1Mn–2Cu/SAPO	459	0.230	1.90	1.054	2.016	0.51	1.587
2Mn–2Cu/SAPO	430	0.228	2.53	2.108	1.998	1.055	2.594
4Mn–2Cu/SAPO	426	0.206	1.96	3.846	2.048	1.878	3.424
8Mn–2Cu/SAPO	424	0.196	2.54	8.306	1.848	4.495	9.104

a Determined by ICP.

b Determined by XPS.

The manganese loading, copper loading and Mn/Cu molar ratio from ICP result were almost the same as the set values. Meanwhile the Mn/Cu molar ratio from the XPS results was quite different from that of ICP results. Since XPS and ICP reflect the elemental information of the surface phase and the bulk phase respectively, the difference might come from the enrichment of manganese on surface.

### 3.5 XRD

**Fig. 4:**
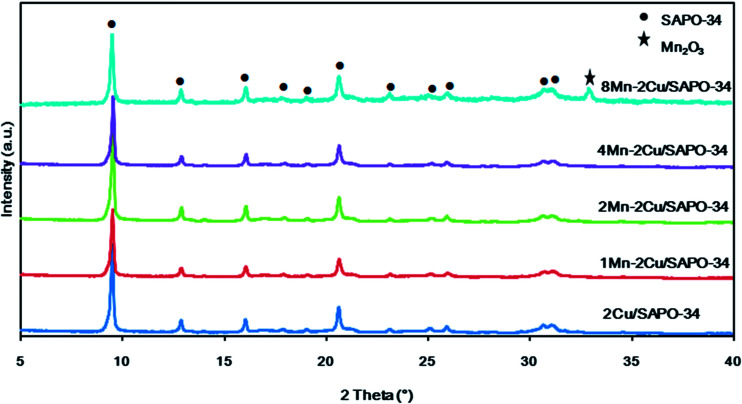
XRD patterns of *x*Mn–2Cu/SAPO.

XRD patterns of *x*Mn–2Cu/SAPO are depicted in Fig. 4. The diffraction peaks of chabazite phase with the space group of *R*3̄*m* were identified, indicating that the crystalline structure of SAPO-34 remained unchanged after catalyst preparation. When manganese loading increased from 0 to 4%, only the chabazite phase was detectable, indicating that the manganese species were well dispersed. As manganese loading reached 8%, the diffraction peaks of the Mn_2_O_3_ phase appeared, indicating the aggregation of manganese species and decreased manganese dispersity. On *x*Mn/SAPO (*x* = 1, 2, 4, 8), which was previously prepared,^14^ there too were no peaks attributed to manganese oxides until manganese loading increased to 8%.

### 3.6 NH_3_-TPD

**Fig. 5:**
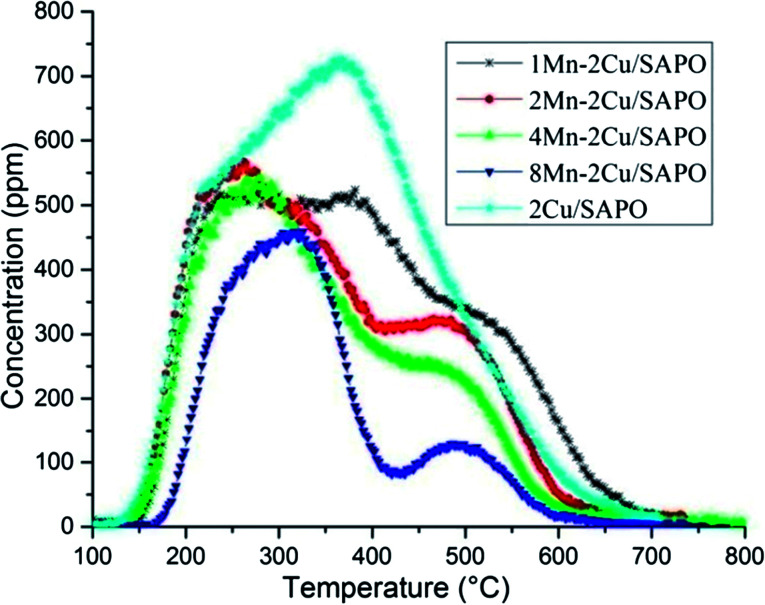
NH_3_-TPD of *x*Mn–2Cu/SAPO.

Fig. 5 showed the effluent NH_3_ profiles during the NH_3_-TPD process. The amount of NH_3_ desorption was closely related to the amount of acid sites. As expected, when manganese loading increased, the amount of NH_3_ desorption decreased, which was caused by the replacement of H^+^ in the framework of SAPO by Mn^*x*+^ in the ion exchange process.

In Fig. 5 there are three NH_3_ desorption peaks, *i.e.* a low temperature peak at 250 °C, a middle temperature peak at 400 °C and a high temperature peak at 500 °C, which could be assigned to the weak acid site, the middle strong acid site and the strong acid site, respectively.^17^

The shapes of 1Mn–2Cu/SAPO, 2Mn–2Cu/SAPO and 4Mn–2Cu/SAPO were similar while the activity differed a lot, indicating that the related acid sites might not have significant influence on the SCR activity for *x*Mn–2Cu/SAPO. For *x*Mn/SAPO (*x* = 1, 2, 4, 8),^14^ the amount of ammonia desorption also decreased as manganese loading increased.

### 3.7 H_2_-TPR

**Fig. 6:**
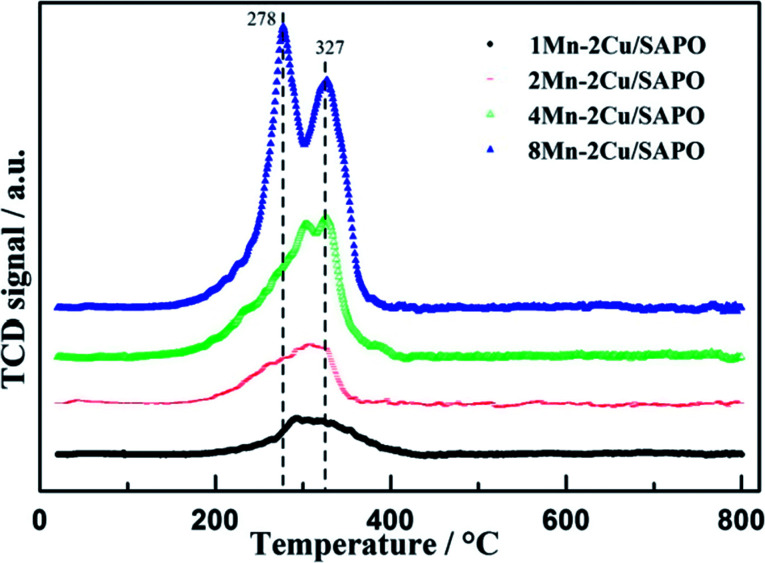
H_2_-TPR of *x*Mn–2Cu/SAPO.

The H_2_-TPR profiles of the four catalysts are depicted in Fig. 6. For 8Mn–2Cu/SAPO, there were two sharp peaks at 278 °C and 327 °C, respectively. The former indicated the reduction of MnO_2_ to Mn_2_O_3_, while the latter indicated the reduction of Mn_2_O_3_ to Mn_3_O_4_.^3^ Compared with Mn/TiO_2_,^18,19^ the reduction peaks moved to a lower temperature, indicating its good redox capacity.

In the TPR patterns, the reduction process of copper in SAPO-34 zeolite could be separated into two steps, *i.e.* the reduction of Cu^2+^ to Cu^+^ at about 300 °C and the reduction of Cu^+^ to Cu^0^ at about 440 °C,^10^ while in Fig. 6 the peak strength of 1Mn–2Cu/SAPO was relatively low, indicating that the peak strength of 2Cu/SAPO was even lower, making the assignation of copper species difficult and unreasonable, so the assignation of copper was not discussed. The hydrogen consumption amount of copper reduction was relatively low, so the ignorance of copper species would not influence the discussion of manganese species.

As manganese loading increased, the peaks at 278 °C and 327 °C increased, indicating that the proportion of Mn^3+^ and Mn^4+^ species increased. For 1Mn–2Cu/SAPO catalysts, only one broad peak between 250 and 400 °C was detectable, indicating that most of the manganese species were Mn^2+^ since Mn^2+^ cannot be reduced below 700 °C. Since Mn^2+^ species were inactive for SCR reaction, addition of manganese did not help increase the activity for 1Mn–2Cu/SAPO, which was consistent with *E*_p_ trends and NO oxidation results. For 2Mn–2Cu/SAPO the peaks belonging to Mn^3+^ and Mn^4+^ species started to generate, indicating its good redox capacity. For 4Mn–2Cu/SAPO and 8Mn–2Cu/SAPO, the peaks at 278 °C and 327 °C increased greatly, indicating that the ratio of Mn^3+^ and Mn^4+^ increased. When manganese loading was above 2%, Mn^3+^ and Mn^4+^ species began to generate, which is consistent with the *E*_p_ trends and NO oxidation results.

Compared with *x*Mn/SAPO (*x* = 1, 2, 4, 8) previously prepared,^14^ for which the temperature peak of H_2_-TPR was about 353 °C and 442 °C, the peak temperature moved to a lower value, indicating that the interactions between copper and manganese species might accelerate oxidation reaction and increase the catalytic oxidation activity, which would promote low temperature SCR reaction.

Table 2 represents the hydrogen consumption of *x*Mn–2Cu/SAPO during TPR processes. As manganese loading increased, hydrogen consumption increased gradually, while the actual hydrogen consumption was much lower than the theoretical value. The ratio of experimental hydrogen consumption to theoretical hydrogen consumption of 8Mn–2Cu/SAPO and 4Mn–2Cu/SAPO was similar, as well as that of 1Mn–2Cu/SAPO and 2Mn–2Cu/SAPO. Since the ratio of experimental hydrogen consumption to theoretical hydrogen consumption was highly related to the proportion of Mn^3+^ and Mn^4+^, the proportion of Mn^3+^ and Mn^4+^ for 1Mn–2Cu/SAPO and 2Mn–2Cu/SAPO were similar, as well as that of 8Mn–2Cu/SAPO and 4Mn–2Cu/SAPO.

**Table 2:** Hydrogen consumption

	8Mn–2Cu/SAPO	4Mn–2Cu/SAPO	2Mn–2Cu/SAPO	1Mn–2Cu/SAPO
Hydrogen consumption^a^ (10^−2^ mmol)	2.04	1.11	0.468	0.327
Hydrogen consumption^b^ (10^−2^ mmol)	3.53	2.08	1.35	0.989
Hydrogen consumption^a^/hydrogen consumption^b^ (%)	57.8	53.4	35.7	33.1

a The actual hydrogen consumption calculated from TPR profiles.

b The theoretical hydrogen consumption, assuming all of the Cu species are Cu^2+^ and reduced to Cu^0^, and all of the Mn species are Mn^4+^ and reduced to Mn^2+^ in TPR process.

### 3.8 DR UV-vis

**Fig. 7:**
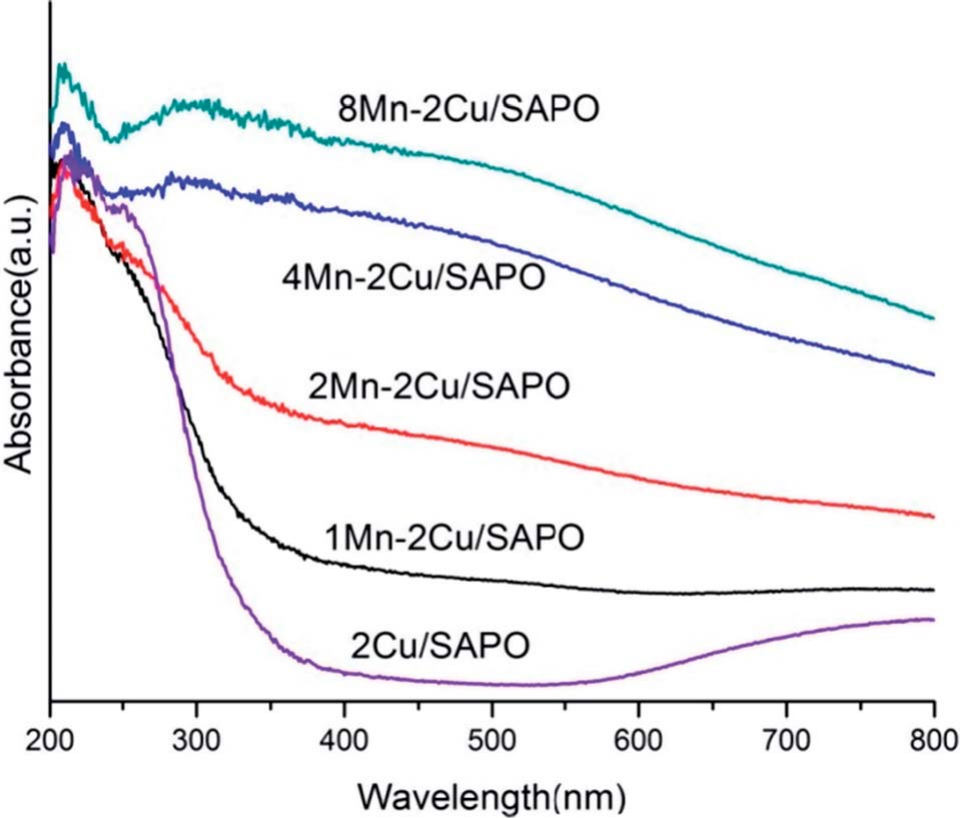
DR UV-vis spectra recorded under ambient atmosphere of *x*Mn–2Cu/SAPO.

The UV-vis spectra of the samples are given in Fig. 7. In Fig. 7, the absorption bands at 240 nm were related to the charge transfer processes between the framework aluminum and oxygen atoms of aluminophosphate.^20^ The band at 220 nm was attributed to the oxygen-to-metal charge-transfer of the isolated Cu^+^/Cu^2+^ bound on the framework of zeolites.^21,22^ The bands at 280–300 nm were assigned to copper oxide clusters.^23,24^ The bands at 700–800 nm were assigned to the d–d transition of isolated Cu^2+^ in weak disordered octahedral coordination of O-containing ligands.^23,25^

The broad bands in the range 320–380 nm were attributed to the Mn^3+^ ← O^2−^ charge transfer transition superimposed on the ^5^B_1g_ → ^5^B_2g_ crystal field d–d transition.^26^ The band at 322 nm was tentatively assigned to the Mn^3+^ ← O^2−^ charge transfer in Mn_3_O_4_ in which manganese was octahedrally coordinated with oxygen.^27–29^ The band at 255–276 nm could be assigned to the Mn^2+^ ← O^2−^ charge transfer transition in tetrahedral oxygen coordination.^27,28^ In the α-Mn_2_O_3_ structure, Mn^3+^ ions occupied octahedral sites and, if highly symmetric, a single spin-allowed absorption band in the d–d transition region was expected similarly to [Mn(H_2_O)_6_]^3+^ at 500 nm.

As manganese loading increased, the band at 320–380 nm increased gradually especially when the manganese loading changed from 2% to 4%, indicating that the percentage of Mn^3+^ increased.

Meanwhile the bands at 255–276 nm were generated, indicating that the percentage of Mn^2+^ decreased. Consistent with the H_2_-TPR result, the UV-vis results showed the partial oxidation of Mn^2+^ to Mn^3+^ or Mn^4+^_,_ when manganese loading increased.

The UV-vis spectra of *x*Mn–2Cu/SAPO were similar to that of *x*Mn/SAPO (*x* = 1, 2, 4, 8)^14^ in that the percentage of Mn^3+^ increased suddenly as manganese loading increased from 2% to 4%, indicating that the distribution of manganese species might not have been influenced by the copper species.

### 3.9 XPS

**Fig. 8:**
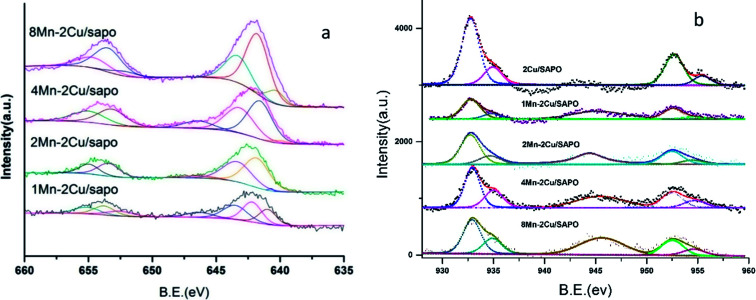
XPS spectra of (a) Mn 2P and (b) Cu 2P.

Fig. 8a shows the XPS spectra of Mn 2P of *x*Mn–2Cu/SAPO. Two main peaks assigned respectively to Mn 2P_3/2_ at 642.5 eV and Mn 2P_1/2_ at 654 eV were observed.

By performing peak-fitting deconvolution, the Mn 2P_3/2_ peak could be separated into three peaks, *i.e.* 640.8–640.9 eV, 642.1–642.3 eV and 643.8–644.1 eV, which correspond to the Mn^2+^ species, Mn^3+^ species and Mn^4+^ species,^3,30^ respectively. The atomic percentages of manganese species in different valence states are shown in Table 3.

**Table 3:** Binding energies (eV) of Mn^*x*+^ 2P_3/2_ and the percentage of Mn^*x*+^

Compound	Mn^2+^		Mn^3+^		Mn^4+^	
	Peak (eV)	%	Peak (eV)	%	Peak (eV)	%
1Mn–2Cu/SAPO	640.97	26.3	642.19	38.8	643.51	34.9
2Mn–2Cu/SAPO	640.69	0.5	641.83	55.8	643.36	44.2
4Mn–2Cu/SAPO	639.99	0.1	641.58	56.2	643.18	43.8
8Mn–2Cu/SAPO	640.42	9.0	641.83	60.7	643.44	30.3

When manganese loading increased from 1% to 2%, the percentage of Mn^3+^ and Mn^4+^ increased and the percentage of Mn^2+^ decreased a lot, while when manganese loading kept increasing, the percentage of manganese species did not change much. This was consistent with the NO oxidation, H_2_ TPR and UV-vis results.

As manganese loading increased, the percentage of Mn^3+^ species increased, and the percentage of Mn^4+^ species also increased, except for 8Mn–2Cu/SAPO. This was consistent with the UV-vis and H_2_-TPR results. Though H_2_-TPR, UV-vis and XPS all showed that there were many Mn^4+^ and Mn^3+^ species on 8Mn–2Cu/SAPO, the XRD patterns showed the Mn_2_O_3_ phase, which indicated the aggregation of manganese species and decreased manganese dispersity, and the NH_3_-TPD results showed that there were much less acid sites. The decreased manganese dispersity and the less acid sites could lead to the lower SCR activity. The XPS spectra of *x*Mn/SAPO (*x* = 1, 2, 4, 8)^14^ also showed that most of the manganese species were Mn^3+^ and Mn^4+^.

Fig. 8b shows the XPS spectra of Cu 2P; two peaks respectively attributed to Cu 2P_3/2_ at 932.6 eV and Cu 2P_1/2_ at 952.7 eV were observed. The shake-up satellite was found at about 945 eV, indicating the presence of Cu^2+^ species. As manganese loading increased, the shake-up satellite increased, indicating that the amount of Cu^2+^ species increased.

By performing peak-fitting deconvolution, the Cu 2P_3/2_ peak could be separated into two peaks, at about 932.7 eV and 934.8 eV respectively, which was ascribed to the Cu^+^ and Cu^2+^ species.^31^ The atom percentage of Cu^+^ and Cu^2+^ is shown in Table 4. As manganese loading increased, the ratio of Cu^2+^ increased, indicating that the addition of manganese might oxidize Cu^+^ into Cu^2+^, which was strong evidence that there was strong interaction between the copper and manganese species.

**Table 4:** Binding energies (eV) of Cu^*x*+^ 2P_3/2_ and the percentage of Cu^*x*+^

Compound	Cu^+^		Cu^2+^	
	Peak (eV)	%	Peak (eV)	%
2Cu/SAPO	932.75	78.3	934.93	21.7
1Mn–2Cu/SAPO	932.75	79.9	934.81	20.1
2Mn–2Cu/SAPO	932.64	75.6	934.56	24.4
4Mn–2Cu/SAPO	932.84	65.7	934.93	34.3
8Mn–2Cu/SAPO	932.82	67.2	934.80	32.7

**Fig. 9:**
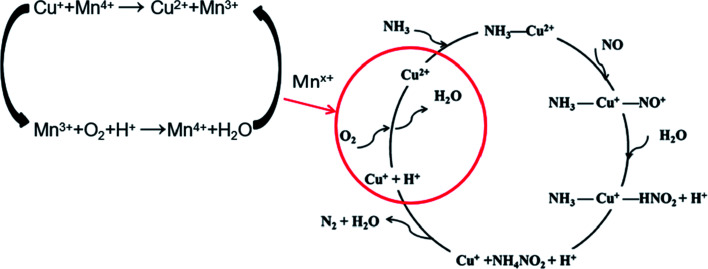
The proposed low temperature SCR mechanism on 2Mn–2Cu/SAPO.

According to the results above and referring to the mechanism of SCR on Cu/SAPO Feng Gao^9^ had put forward, the mechanism of SCR on 2Mn–2Cu/SAPO could be proposed. Fig. 9 showed the proposed low temperature SCR mechanism on 2Mn–2Cu/SAPO. In the catalytic cycle, Mn^*x*+^ acts as a catalyst for the oxidation step of Cu^+^ to Cu^2+^ in low temperature SCR reaction.

### 3.10 DRIFTS

**Fig. 10:**
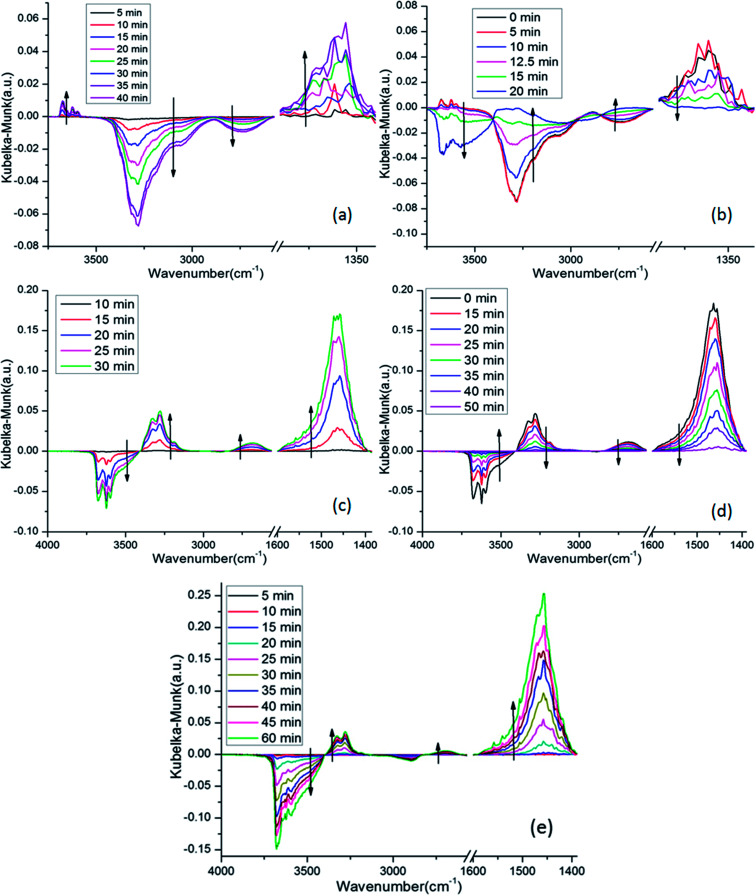
DRIFTS spectra of 4Mn–2Cu/SAPO at 150 °C exposed to gas phase of (a) 500 ppm NO + 3% vol O_2_ + N_2_ (balance); (b) 500 ppm NH_3_ + 3% vol O_2_ + N_2_ (balance) after saturation with NO; (c) 500 ppm NH_3_ + 3% vol O_2_ + N_2_ (balance); (d) 500 ppm NO + 3% vol O_2_ + N_2_ (balance) after saturation with NH_3_; (e) 500 ppm NO + 500 ppm NH_3_ + 3% vol O_2_ + N_2_ (balance).

*In situ* DRIFTS was performed to identify the adsorbed NO species. Fig. 10 shows the DRIFTS spectra of 4Mn–2Cu/SAPO at 150 °C exposed to different gas phases. In Fig. 10a, there was only one positive band at 1360 cm^−1^ in the range of 1200–1600 nm, which belongs to nitrite groups,^32,33^ suggesting that those NO species were nitrite groups. The negative bands at 3280 cm^−1^ and 2730 cm^−1^ might be attributed to the depletion of Brønsted base sites by the nitrite groups. Three bands at 3675 cm^−1^, 3623 cm^−1^ and 3598 cm^−1^ could be attributed to the appearance of O–H bond of nitrite groups (–N–O–H) in different chemical environments. When NH_3_ was introduced into the system, as shown in Fig. 10b, the bands associated with nitrite groups decreased rapidly, indicating reaction between NH_3_ and NO related species.

In Fig. 10c, when NH_3_ was introduced into the system, the bands at 1463 cm^−1^ increased greatly, indicating the generation of NH_3_ species on the Brønsted acid sites,^22^ while the bands at 1210 cm^−1^ increased slightly, which was consistent with the fact that most of the acid sites on SAPO-34 were Brønsted acid sites. The negative bands at 3681 cm^−1^, 3625 cm^−1^ and 3600 cm^−1^ were attributed to the depletion of Brønsted acid sites, which was caused by the adsorption of NH_3_. Specifically, the negative bands at 3681 cm^−1^ were assigned to the occupation of P–OH sites by NH_3_. The other two bands were related to the depletion of Si–OH–Al acid sites. The band at 3147–3396 cm^−1^ was attributed to the N–H stretching vibrations of NH_4_^+^.^22^ When NO was introduced into the system, as shown in Fig. 10d, the bands related to the NH_3_ species decreased gradually, which was caused by the reaction between NO and NH_3_.

Fig. 10e and c are similar in that only bands related to NH_3_ are visible, while no other bands appeared, especially the negative bands at 2700 cm^−1^ and 3000–3400 cm^−1^ were invisible.

This was strong evidence that only the Elay–Rideal mechanism was effective on the 4Mn–2Cu/SAPO at 150 °C. Since if Langmuir–Hinshelwood mechanism was effective, the adsorbed and well activated NO and NH_3_ related species would react with each other, while the NO species not well activated would accumulated and be detectable for DRIFTS, leading to the appearance of negative bands at 2700 and 3000–3400 nm^−1^, as Fig. 10a and b show. Due to the fact that those peaks were not observed in Fig. 10e, all these observations lead to the exclusion of the Langmuir–Hinshelwood mechanism and the assurance of the Elay–Rideal mechanism. In the Elay–Rideal mechanism, NO molecules from gas phase directly react with the well activated NH_3_ species adsorbed on the surface and leave as products to gas phase with the active site left.

## 4 Conclusion

In this work, the SCR activity and physicochemical properties of *x*Mn–2Cu/SAPO were investigated. The H_2_-TPR, UV-vis and XPS results showed that Mn^4+^ and Mn^3+^ species began to appear when manganese loading reached 2%. The NO oxidation results showed that 2Mn–2Cu/SAPO had the best NO oxidation activity, indicating that good interaction existed between copper and manganese, which was consistent with the XPS results of Cu 2p binding energy.

The graph of the promotion effect of manganese showed that for 2Mn–2Cu/SAPO, manganese had a good promotion effect on 2Cu/SAPO. According to H_2_-TPR, UV-vis, XPS and NO oxidation results, the promotion effect of manganese on 2Cu/SAPO comes from the generation of Mn^3+^ and Mn^4+^ species. The addition of manganese increased the ratio of Cu^2+^. The Mn^3+^ and Mn^4+^ species might take part in the catalytic cycle and accelerate the oxidation of Cu^+^ to Cu^2+^.

From the *in situ* DRIFTS results, the acid sites on the surface were mainly Brønsted acid sites, the main mechanism on 4Mn–2Cu/SAPO-34 at low temperature was the Elay–Rideal mechanism.

## Conflicts of interest

There are no conflicts to declare.

## Supplementary Material
